# Blocking the association of HDAC4 with MAP1S accelerates autophagy clearance of mutant Huntingtin

**DOI:** 10.18632/aging.100818

**Published:** 2015-10-24

**Authors:** Fei Yue, Wenjiao Li, Jing Zou, Qi Chen, Guibin Xu, Hai Huang, Zhen Xu, Sheng Zhang, Paola Gallinari, Fen Wang, Wallace L. McKeehan, Leyuan Liu

**Affiliations:** ^1^ Institute of Biosciences and Technology, Texas A&M Health Science Center, Houston, TX 77030, USA; ^2^ Department of Urology, The Fifth Affiliated Hospital of Guangzhou Medical University, Guangzhou, Guangdong Province, China; ^3^ Department of Urology, The Sun Yat-sen Memorial Hospital, Sun Yat-sen University, Guangzhou, China; ^4^ The Brown Foundation Institute of Molecular Medicine for the Prevention of Human Diseases, The University of Texas Health, Science Center at Houston, Houston, TX 77030, USA; ^5^ Exiris Srl, Rome, Italy; ^6^ Department of Molecular and Cellular Medicine, College of Medicine, Texas A&M Health Science Center, TX 77843, USA

**Keywords:** acetylation, AGERA, aggregate, apicidin, autophagy, C19ORF5, CRISP/Cas9 system, deacetylase, HDAC4, huntingtin, huntington's disease, LC3, MAP1S, N2a, stability

## Abstract

Autophagy controls and executes the turnover of abnormally aggregated proteins. MAP1S interacts with the autophagy marker LC3 and positively regulates autophagy flux. HDAC4 associates with the aggregation-prone mutant huntingtin protein (mHTT) that causes Huntington's disease, and colocalizes with it in cytosolic inclusions. It was suggested HDAC4 interacts with MAP1S in a yeast two-hybrid screening. Here, we found that MAP1S interacts with HDAC4 via a HDAC4-binding domain (HBD). HDAC4 destabilizes MAP1S, suppresses autophagy flux and promotes the accumulation of mHTT aggregates. This occurs by an increase in the deacetylation of the acetylated MAP1S. Either suppression of HDAC4 with siRNA or overexpression of the MAP1S HBD leads to stabilization of MAP1S, activation of autophagy flux and clearance of mHTT aggregates. Therefore, specific interruption of the HDAC4-MAP1S interaction with short peptides or small molecules to enhance autophagy flux may relieve the toxicity of mHTT associated with Huntington's disease and improve symptoms of HD patients.

## INTRODUCTION

Mammalian histone deacetylases (HDAC) are lysine deacetylases, which are classified into three main groups based on their homology to yeast proteins.

HDAC4 belongs to group II subgroup A of the family [1]. Huntington's disease (HD) is a fatal progressive neuro-degenerative disorder caused by an autosomal dominant mutation with expansion of more than 36 trinucleotide CAG repeats (which codes for polyglutamine) in exon 1 of the *huntingtin* (*HTT*) gene that encodes huntingtin (HTT) protein [2]. HDAC4 associates with the mutant HTT (mHTT) and colocalizes with it in cytoplasmic inclusions [3]. In mouse models of Huntington's disease, HDAC4 reduction delays cytoplasmic formation of mHTT aggregates and rescues neuronal and cortico-striatal synaptic function, but does not repair the global transcriptional dysfunction [3]. However, the mechanism by which HDAC4 reduction delays cytoplasmic formation of mHTT aggregates is unknown.

Autophagy is a process that begins with the formation of isolation membranes that recognize and engulf substrates such as aggregated proteins to form autophagosomes. These autophagosomes migrate along acetylated microtubules and fuse with lysosomes to generate autolysosomes in which autophagosomal cargos are degraded [4, 5]. Defects in autophagy in neurons cause accumulation of aggregate-prone proteins such as mHTT whose toxicity results in neurodegeneration [6]. Microtubule-associated protein 1S (MAP1S, previously called C19ORF5) associates with microtubules [7, 8]. Like its sequence homologues MAP1A and MAP1B, MAP1S interacts with mammalian autophagy marker LC3 [9–11], and bridges components involved in autophagy with microtubules to affect autophagosomal biogenesis and degradation [11]. Depletion of MAP1S results in decreased levels of Bcl-2 and P27 and was proposed to reduce initiation of autophagy. Both the direct function of MAP1S association with microtubules through LC3 and indirect positive impact of MAP1S on autophagy initiation through Bcl-2 and P27 affect the overall rate of autophagy flux [11]. A potential interaction between HDAC4 and MAP1S revealed in a yeast two-hybrid screen [12] triggered us to investigate whether HDAC4 regulates autophagy to reduce mHTT aggregates via modification of MAP1S.

We discovered that MAP1S interacts with HDAC4 through a HDAC4-binding domain (HBD) within the overlapping region between the short chain (SC) and the heavy chain (HC) of MAP1S. MAP1S had no effect on levels and subcellular distribution of HDAC4. However, HDAC4 decreased the stability of MAP1S by catalyzing deacetylation of acetylated MAP1S and further led to suppression of autophagy flux and accumulation of mHTT aggregates. Inhibition of HDAC4 or overexpression of HBD promoted stabilization of MAP1S and restored the MAP1Sregulated autophagy flux and degradation of mHTT aggregates. This reveals a new potential to treat Huntington's disease by interrupting the specific interaction between HDAC4 and MAP1S.

## RESULTS

### Inhibition of HDAC4 ameliorates aggregation of mHTT

**Figure 1:**
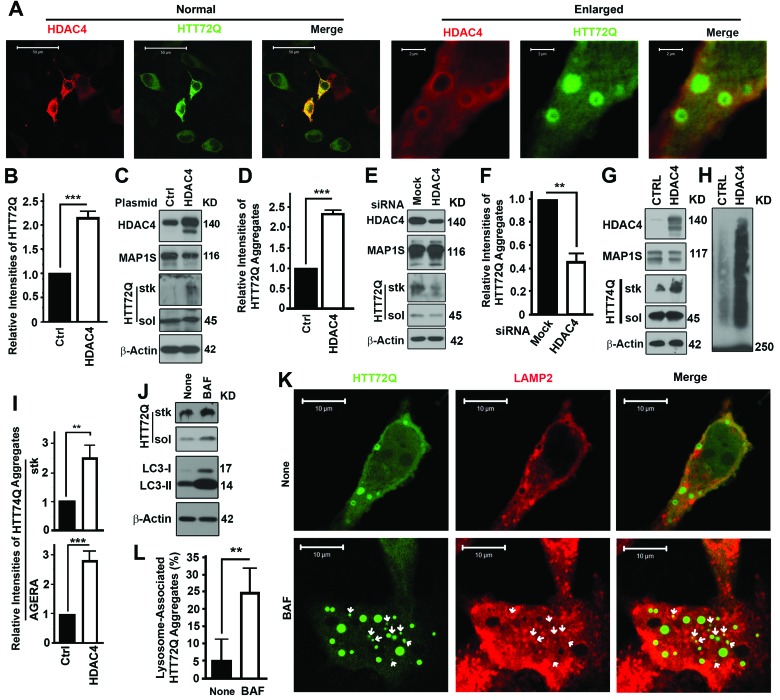
Inhibition of HDAC4 reduces mHTT aggregates (**A-D**) Overexpression of HDAC4 increases levels of GFP-HTT72Q (HTT72Q) in HeLa cells stably expressing HTT72Q. Representative fluorescent images (**A**) and immunoblot results (**C**) and their respective quantification (**B,D**) are shown. Bars in (A) were 50 or 2 μm in the normal view on the left half and enlarged view on the right. HTT72Q aggregates retained in stacking gel (stk) and soluble HTT72Q (sol) were labeled. Data here or throughout are the average ± standard deviation of at least three repeats. Statistical significance was determined by Student's *t*-test. *, *p* ≤ 0.05; **, *P* ≤ 0.01; and ***, *P* ≤ 0.001. The 45 KD HTT72Q formed aggregates that failed to penetrate stacking gel. (**E, F**) Suppression of HDAC4 decreases levels of HTT72Q aggregates in HeLa cells stably expressing HTT72Q. Representative results of immunoblots (**E**) and quantification (**F**) when HDAC4 is suppressed with siRNA are shown. (**G-I**) Overexpression of HDAC4 increases levels of GFP-HTT74Q (HTT74Q) in N2a cells transiently expressing HTT74Q. Representative results from normal immunoblot analyses of aggregates in stacking gel (**G**) or AGERA (**H**) and their respective quantification (**I**) were shown. (**J**) Lysosomal inhibitor Bafilomycin A1 (BAF) causes accumulation of both HTT72Q and LC3-II in cells expressing HTT72Q. None, without BAF. (**K, L**) HTT72Q aggregates colocalize with LAMP2-labelled lysosomes (red) in cells stably expressing HTT72Q and transiently expressing Flag-HDAC4 in the presence of BAF. Representative fluorescent images are shown and white arrows indicate HTT72Q aggregates that colocalize with LAMP2 (**K**). Statistical significance of difference in the percentages of HTT72Q aggregates associated with LAMP2-labelled lysosomes to total aggregates was assessed between control and BAF-treated cells (**I**).

GFP-HTT72Q, a GFP-tagged mHTT variant, includes a polypeptide encoded by exon 1 of the *Huntingtin* gene plus 72 expanded polyglutamine [poly(Q)] repeats in the N-terminus [13, 14]. The acetylation of huntingtin at residue K444 promotes autophagic degradation of huntingtin itself [15]. The K444 residue is out of the sequence covered by HTT72Q so that HTT72Q degradation is not affected by the acetylation of K444. Overexpression of HDAC4 in cells expressing GFPHTT72Q led to enhancement of GFP-HTT72Q fluorescence (Figure 1A,B) and levels of GFP-HTT72Q aggregates (Figure 1C,D). Increasing levels of HDAC4 distributed surrounding the HTT72Q aggregates (Figure 1A). Suppression of HDAC4 levels with HDAC4-specific siRNA led to reduction of mHTT aggregates (Figure 1E,F). Analyses with Agarose Gel Electrophoresis for Resolving Aggregates (AGERA) revealed the same results as in the normal immunoblot analyses of aggregates in stacking gel. Accumulation of another mHTT (GFP-HTT74Q) with a similar role as the GFPHTT72Q in Huntington's disease was observed when the GFP-HTT74Q and HDAC4 were transiently coexpressed in neuroblastoma Neuro-2a (N2a) cells (Figure 1G-I). Thus, the inhibition of HDAC4 greatly reduces the severity of aggregation of mHTT.

### HDAC4 inhibition enhances autophagy flux

Consistent with a previous report [16], GFP-HTT72Q was degraded in lysosomes. Inhibition of lysosomal activity with Bafilomycin A1 (BAF) led to accumulation of mHTT aggregates together with the autophagic marker LC3-II (Figure 1J). The percentage of GFP-HTT72Q aggregates that overlapped with LAMP2-labeled lysosomes was significantly increased in the presence of BAF (Figure 1K,L). This suggested that small aggregates were efficiently degraded through lysosomes in the absence of Bafilomycin A1, but large aggregates accumulated because of the compromised lysosomal degradation.

**Figure 2:**
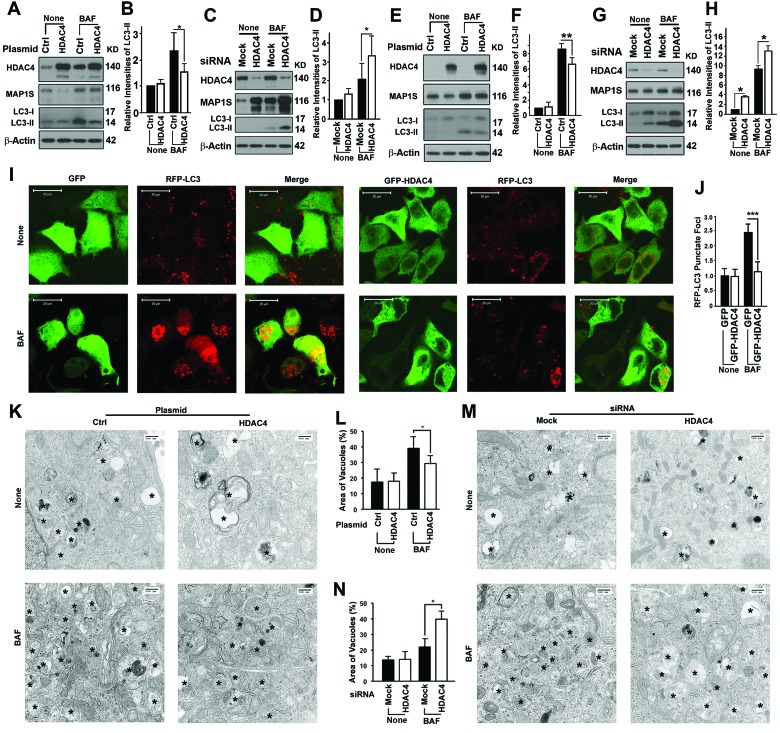
HDAC4 inhibits autophagy flux (**A-D**) HDAC4 affects levels of MAP1S and LC3-II in HeLa cells in the absence (None) or presence of BAF. Representative immunoblot results (**A, C**) and their respective quantification (**B, D**) of the impact of HDAC4 overexpression (**A, B**) and HDAC4 suppression with siRNA (**C, D**) are shown. (**E-H**) HDAC4 affects levels of MAP1S and LC3-II in N2a cells in the absence (None) or presence of BAF. Representative immunoblot results (**E, G**) and their respective quantification (**F, H**) of the impact of HDAC4 overexpression (**E, F**) or HDAC4 suppression with siRNA (**G, H**) are shown. (**I, J**) HDAC4 overexpression reduces punctate foci of RFP-LC3 in HeLa cells stably expressing RFP-LC3. Fluorescence microscopy images (**I**) and quantification (**J**) of punctate foci of RFP-LC3 in the absence or presence of BAF are shown. (**K-N**) HDAC4 overexpression (**K, L**) or siRNA suppression (**M, N**) alters the vacuolar areas in HeLa cells. Transmission electron microscopic images (**K, M**) and quantification (**L, N**) are shown. Symbol “*” indicates vacuoles.

We then tested whether inhibition of HDAC4 enhanced autophagy that would consequently promote degradation of mHTT aggregates. Overexpression of HDAC4 led to a reduction in levels of MAP1S, an enhancer of autophagy flux [11]. Impairment of autophagy flux due to the HDAC4 overexpression was confirmed by reduced levels of LC3-II in HeLa cells in the presence of BAF (Figure 2A,B). Suppression of HDAC4 with siRNA (Figure 2C,D) resulted in an increase in levels of MAP1S. This was accompanied by increased levels of LC3-II in the presence of BAF. The HDAC4 overexpression-triggered impairment of autophagy flux and HDAC4 suppression-induced activation of autophagy flux were further confirmed in N2a cells (Figure 2E-H). Autophagy flux measured at the cellular level by punctate foci of RFP-LC3 observed by fluorescent microscopy was impaired by HDAC4 overexpression (Figure 2I,J). Autophagy flux measured by autophagy vacuoles observed under the electron microscope was impaired by HDAC4 overexpression (Figure 2K,L) or enhanced by HDAC4 suppression (Figure 2M,N). Interestingly, the vacuoles in the MAP1S suppressed cells in the presence of BAF relatively contained less substrate contents (Figure 2M), indicating a deficiency in autophagy cargos when initiation of autophagy is exceedingly activated as others described [17]. Thus, HDAC4 activity inhibits autophagy flux and inhibition of the activity enhances autophagy flux.

### HDAC4 regulation of autophagy-mediated degradation of mHTT depends on MAP1S

**Figure 3:**
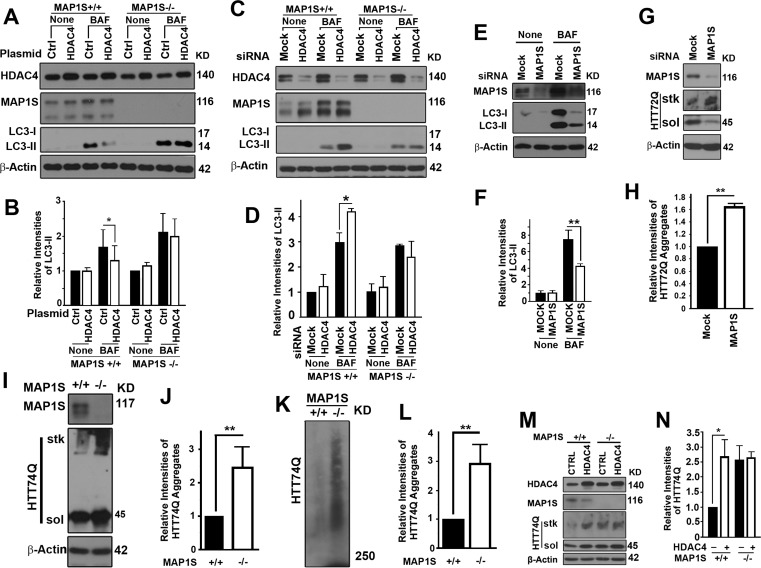
HDAC4 inhibits MAP1S-mediated autophagy clearance of mHTT aggregates (**A-D**) MAP1S is required for HDAC4 to affect LC3-II. Representative immunoblot results (**A, C**) and quantification (**B, D**) of the impact of MAP1S deletion on the effect of HDAC4 overexpression in wild-type and MAP1S−/− MEF cells (**A, B**) or suppression with siRNA in wild-type and MAP1S−/− HeLa cells (**C, D**) on LC3-II in the absence or presence of BAF. (**E, F**) MAP1S suppression with siRNA reduces levels of LC3-II in cells stably expressing HTT72Q. Representative immunoblots (**E**) and their quantification (**F**) are shown. (**G, H**) MAP1S suppression with siRNA increases levels of HTT72Q aggregates in cells stably expressing HTT72Q. Representative immunoblots (**G**) and their quantification (**H**) are shown. (**I-L**) MAP1S depletion increases levels of HTT74Q aggregates in MEF cells transiently expressing GFP-HTT74Q (HTT74Q). Representative immunoblot (**I, K**) and quantification (**J, L**) of the levels of HTT74Q aggregates in wild-type and MAP1S−/− MEF cells analyzed by stack gel (**I, J**) or AGERA (**K, L**). (**M, N**) MAP1S is required for the HDAC4-dependent increases in levels of HTT74Q aggregates. Representative immunoblots (**M**) and quantification (**N**) of the differences between wild-type and MAP1S−/− MEF cells are shown.

To determine whether the regulation of autophagy flux by HDAC4 depended on MAP1S, we enhanced the expression of HDAC4 in wild-type and MAP1S^−/−^ MEF cells or suppressed the expression of HDAC4 with siRNA in wild-type and MAP1S^−/−^ HeLa cells generated with the CRISPR/Cas9 system. We found that impairment of autophagy flux by overexpression of HDAC4 or the activation of autophagy flux by HDAC4 suppression was only evident in wild-type cells where MAP1S was present; and effects of alteration of HDAC4 levels were abrogated in MAP1S^−/−^ cells (Figure 3A-D). Suppression of MAP1S with siRNA in the HeLa cell line stably expressing mHTT led to an inhibition in autophagy initiation (Figure 3E,F), further confirming our previous report that MAP1S impacts autophagy on both autophagy initiation and autophagosome-lysosome fusion [11]. Consequently, the defects in autophagy triggered by silencing MAP1S caused an accumulation of GFP-HTT72Q aggregates (Figure 3G,H). Accumulation of GFP-HTT74Q was also observed in MAP1S^−/−^ MEF cells transiently expressing the GFP-HTT74Q. The MAP1S-deficiency-triggered accumulation of HTT74Q aggregates was confirmed when the same cell lysates were analyzed by AGERA (Figure 3I-L). Enhancement of mHTT aggregation by overexpression of HDAC4 was only observed in wild-type and not in MAP1S^−/−^ MEF cells (Figure 3M,N). These results indicate that HDAC4-mediated suppression of degradation of mHTT aggregates by autophagy is MAP1S-dependent.

### HDAC4 interacts with MAP1S through the HDAC4-binding domain (HBD)

**Figure 4:**
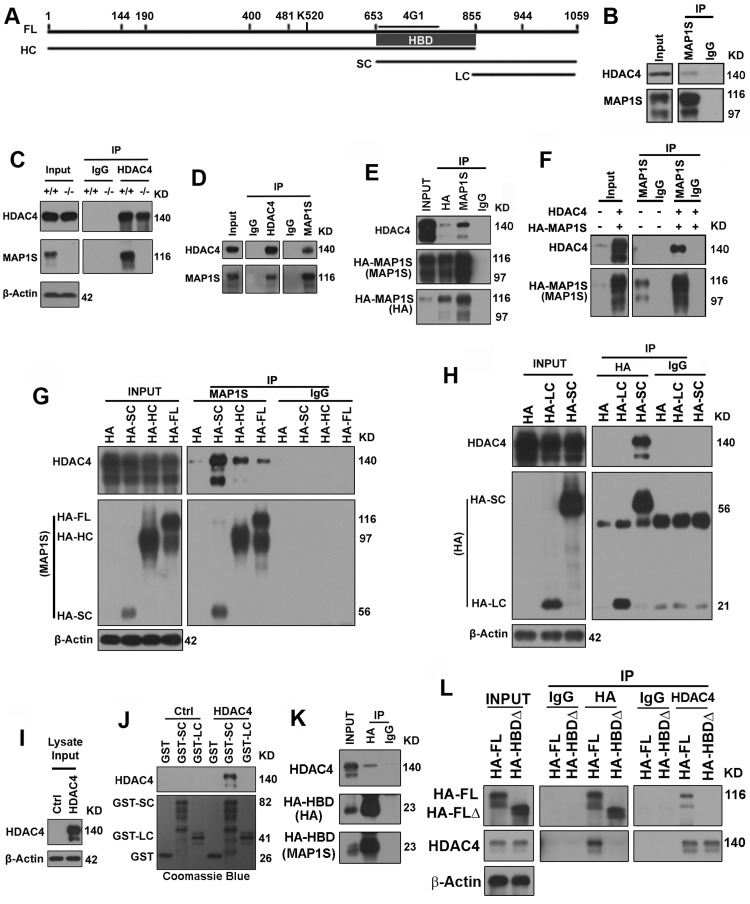
MAP1S interacts with HDAC4 via the HDAC4-binding domain (HBD) in the overlapping region between the HC and SC of MAP1S (**A**) A diagram showing the sequence domains of MAP1S proteins. FL, full length; HC, heavy chain; SC, short chain; LC, light chain; 4G1, region recognized by MAP1S monoclonal antibody 4G1; HBD, HDAC4-binding domain within the R653-Q855 fragment. All sequence numbers were deduced based only on our experimental results. (**B**) Endogenous HDAC4 interacts with MAP1S. HeLa cell lysates were precipitated with a MAP1S-specific antibody 4G1 or IgG control antibody. (**C, D**) Endogenous HDAC4 interacts with MAP1S. Lysates from brain tissues of wild-type (+/+) and MAP1S−/− mice (−/−) (**C**) or N2a cells (**D**) were precipitated with a specific antibody against HDAC4 or MAP1S, or IgG control antibody. (**E, F**) Interaction of HDAC4 and MAP1S in cells overexpressing both HDAC4 and MAP1S FL. In (**E**) lysates from 293T cells overexpressing HA-MAP1S were precipitated with HA or MAP1S-specific antibody or IgG control. In (**F**) lysates from normal 293T cells and 293T cells overexpressing both HDAC4 and HA-MAP1S were precipitated with MAP1S-specific antibody or IgG control. (**G**) HDAC4 interacts with MAP1S isoforms SC, HC and FL. Lysates from 293T cells overexpressing HDAC4 and HA-fused MAP1S isoforms were precipitated with MAP1S-specific antibody or IgG control. (**H**) HDAC4 interacts with MAP1S SC but not LC. Lysates from 293T cells overexpressing HDAC4 and HA-fused MAP1S SC and LC were precipitated with a HA-specific antibody or IgG control. (**I, J**) HDAC4 in cell lysates (**I**) is pulled down by GSH beads bound with purified GST-fused MAP1S SC, but not LC (**J**). HDAC4 was revealed on blots with specific antibody and GST fusion proteins were visualized with Coomassie Blue staining. (**K**) HDAC4 interacts with an overlapping domain between MAP1S HC and SC. Lysates from 293T cells overexpressing HDAC4 and HA-fused MAP1S sequence R653-Q855 (HA-HBD) were precipitated with a HA-specific antibody or IgG control and blotted with HA or MAP1S-specific antibody. (**L**) The Interaction of MAP1S with HDAC4 is abolished when the HBD is deleted. Lysates from 293T cells overexpressing HDAC4 and HA-fused full length MAP1S (HA-FL) or mutant with sequence R653-Q855 deleted (HA-HBDΔ) were precipitated with a HA or HDAC4-specific antibody or IgG control and blotted with HA and HDAC4 antibodies.

Full-length MAP1S (FL) is processed by posttranslational modification to multiple isoforms that include heavy chain (HC), short chain (SC) and light chain (LC) (Figure 4A) [7, 11, 18]. Using a MAP1Sspecific monoclonal antibody 4G1 for immunoprecipitation, we detected complexes of endogenous HDAC4 and MAP1S in HeLa cells (Figure 4B). Further examination of the interaction in brain tissue lysates from wild-type and MAP1S^−/−^ mice and N2a cells revealed that HDAC4 was co-precipitated with MAP1S (Figure 4C,D). The interaction was characterized in more detail with either HA- or MAP1S-specific antibody in 293T cells transiently overexpressing both HDAC4 and HA-tagged MAP1S (HA-MAP1S) (Figure 4E,F). Interaction of HDAC4 with MAP1S HC and SC products in addition to FL was apparent (Figure 4G). This suggested that a domain located between R653 and Q855 (Figure 4A) was necessary and sufficient for the interaction between HDAC4 and MAP1S. This is the region of overlap between HC and SC. MAP1S LC, which lacks this domain, exhibited no interaction with HDAC4 (Figure 4H). The isoform-specific interaction was further confirmed using purified MAP1S SC and LC variants tagged with GST (GST-SC or GST-LC) to pull down HDAC4 from lysates of 293T cells overexpressing HDAC4. Notably, the GST-SC pulled down HDAC4, but GST-LC did not (Figure 4I,J). Using the HA-tagged R653-Q855 fragment (HA-HBD) alone further indicated HBD located within the fragment (Figure 4K). Deletion of HBD in MAP1S led to the abolishment of its interaction with HDAC4 (Figure 4L). Taken together these results indicate that MAP1S interacts with HDAC4 within cells and the interaction occurs through HBD.

### HDAC4 decreases the stability of MAP1S protein

**Figure 5:**
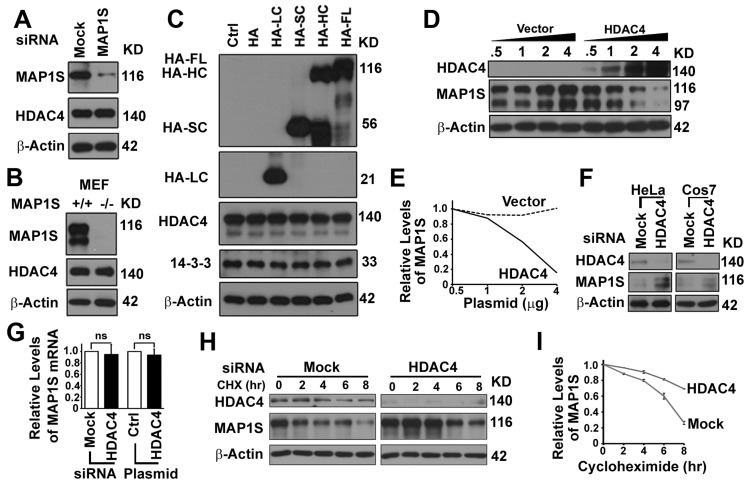
HDAC4 decreases the stability of MAP1S protein (**A, B**) MAP1S depletion has no effect on HDAC4 levels in HeLa cells treated with MAP1S-specific siRNA (**A**) or MEF cells derived from wild-type or MAP1S−/− mice (**B**). (**C**) Overexpression of MAP1S isoforms has no impact on levels of HDAC4 in HeLa cells. (**D, E**) Increasing expression of HDAC4 causes dose-dependent reduction in levels of MAP1S in 293T cells. Representative immunoblots (**D**) and quantification (**E**) are shown. (**F**) HDAC4 depletion with siRNA increases levels of MAP1S in HeLa or COS7 cells. (**G**) HDAC4 suppression or overexpression does not alter levels of MAP1S mRNA. The relative levels of MAP1S mRNA in HeLa cells transfected with siRNA to suppress the expression of HDAC4 or with plasmid for HDAC4 overexpression. (**H, I**) HDAC4 depletion increases the stability of MAP1S proteins. HeLa cells were treated with random (Mock) or HDAC4-specific siRNA (HDAC4), and cellular protein translation was then terminated with cycloheximide (CHX). Samples were collected at different times after cycloheximide treatment. Representative immunoblots (H) and quantification (I) are shown.

To assess potential function of the HDAC4-MAP1S interaction, we tested the effect of cellular levels of one on the other. The siRNA-mediated knockdown of MAP1S in HeLa cells, transgenic deletion of MAP1S in MEF cells, or overexpression of MAP1S isoforms in HeLa cells had no obvious impact on the levels of HDAC4 (Figure 5A-C). Transfection of 293T cells with increasing amounts of HDAC4 plasmid caused a dose-dependent decrease in MAP1S levels (Figure 5D,E). In contrast, suppression of HDAC4 expression with siRNA caused an elevation of MAP1S levels in both HeLa and COS7 cells (Figure 5F). HDAC4-dependent changes in levels of MAP1S occurred strictly on levels of the protein. No change in *MAP1S* mRNA levels was observed (Figure 5G). MAP1S stability was increased when HDAC4 was suppressed (Figure 5H,I). This and the sequence-specific direct interaction of HDAC4 with MAP1S suggests HDAC4 may control levels of MAP1S protein by direct modifications affecting stability.

### HDAC4 decreases levels of acetylated MAP1S

**Figure 6:**
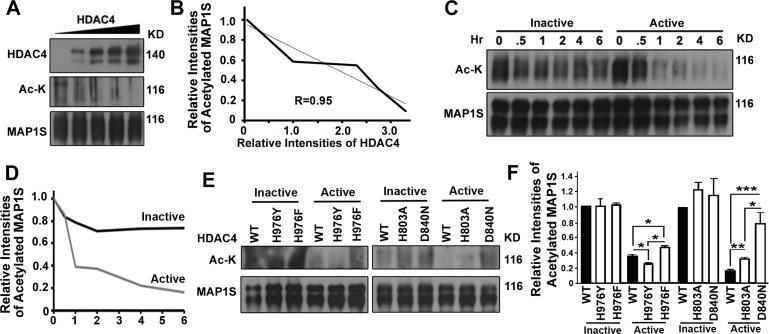
HDAC4 decreases levels of acetylated MAP1S (**A-D**) HDAC4 reduces levels of acetylated MAP1S. HDAC4 was purified from 293T cells overexpressing HDAC4 by immunoprecipitation with antibody against HDAC4; purified HDAC4 (Active) was inactivated by boiling (Inactive). MAP1S purified from MAP1S overexpressing cells with MAP1S antibody was incubated with increasing amounts of active HDAC4 for 2 hrs (**A, B**) or same amount of HDAC4 for increasing amounts of time (**C,D**). Representative immunoblots (**A, C**) and quantification (**B, D**) are shown. (**E, F**) HDAC4 mutants with increased or reduced activity exhibit reduced or enhanced levels of acetylated MAP1S, respectively. HDAC4 mutant proteins purified in same way as the wildtype were incubated with purified MAP1S for 1 hr. Representative immunoblot results (**E**) and quantification (**F**) are shown.

To demonstrate that HDAC4 activity directly affected degree of acetylation of MAP1S, immunoprecipitates of HA-MAP1S were incubated with immuno-purified active HDAC4. Levels of acetylated MAP1S were inversely proportional to increasing levels of HDAC4 (Figure 6A,B) as well as time of incubation with the enzyme (Figure 6C,D). In contrast, no dramatic decrease in levels of acetylated MAP1S was observed when the immunoprecipitates were incubated with heatinactivated HDAC4 (Figure 6C,D).

We then investigated the degree of MAP1S acetylation when incubated with gain or loss of function mutants of HDAC4 [19, 20]. As expected, mutant H976Y that has a reported gain in activity relative to wild-type HDAC4 exhibited significantly higher MAP1S-deacetylation activity and lower levels of acetylated MAP1S than wild-type HDAC4. HDAC4 mutant H803A and D840N that have compromised catalytic activity each exhibited significantly lower MAP1S-deacetylation activity and higher levels of acetylated MAP1S than wild-type HDAC4. Unexpectedly, incubation of MAP1S with the H976F mutant resulted in decreased deacetylation and increased levels of acetylated MAP1S relative to wildtype HDAC4 (Figure 6I,J). The H976F mutant has been reported to be similar to wild-type HDAC4 using other substrates. These results show clearly that acetylated MAP1S is regulated by HDAC4.

### The interaction between HDAC4 and MAP1S is required for HDAC4 to exert suppressive roles on MAP1S-mediated autophagy turnover of mHTT aggregates

**Figure 7:**
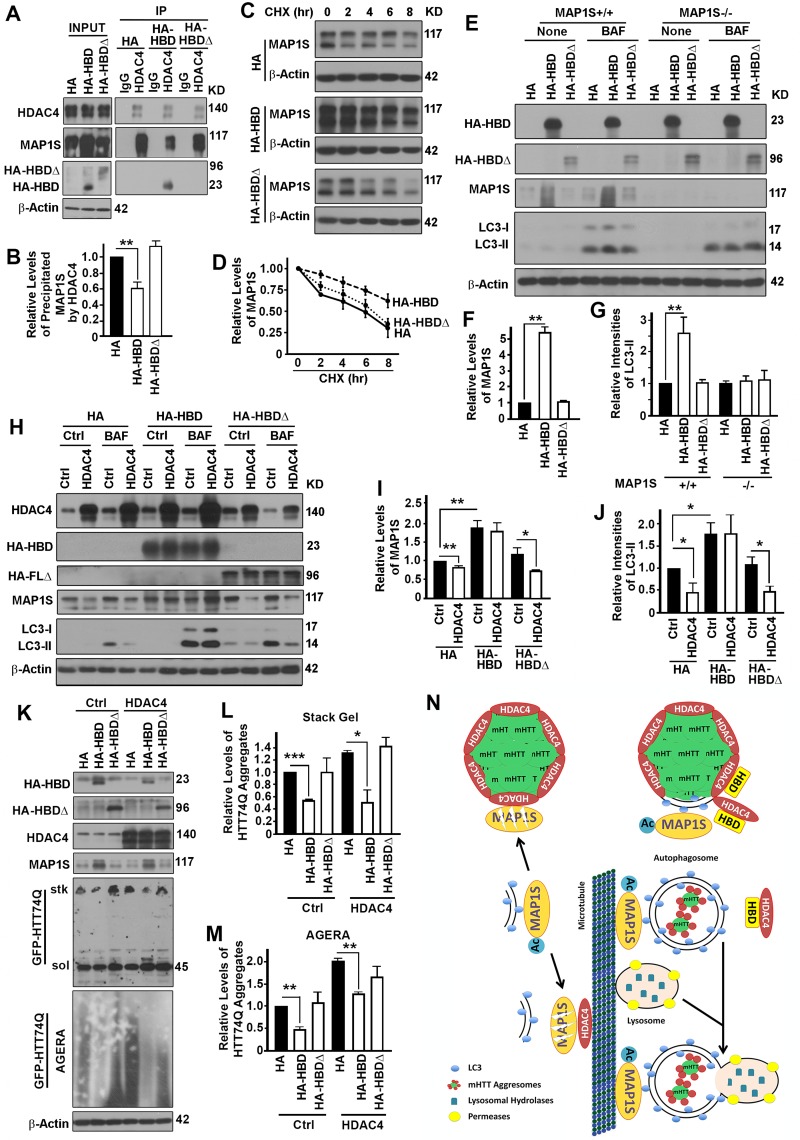
The interaction between HDAC4 and MAP1S is required for HDAC4 to exert suppressive roles on MAP1S-mediated autophagy turnover of mHTT aggregates (**A, B**) Overexpression of HBD but not HA-HBDΔ reduces HDAC4-bound endogenous full length MAP1S. Equal amounts of lysates collected from HeLa cells expressing control HA, HBD and HA-HBDΔ were subjected to immunoprecipitation with HDAC4-specific antibody. Representative immunoblots (**A**) and quantification of the precipitated MAP1S (**B**) were shown. (**C, D**) Overexpression of HBD but not HA-HBDΔ enhances stability of endogenous MAP1S protein in HeLa cells. Lysates from HeLa cells transiently transfected with HA vector and HA-HBD and HA-HBDΔ were collected at different times after cycloheximide treatment. Representative immunoblots (**C**) and quantification (**D**) are shown. (**E-G**) Overexpression of HBD but not HA-HBDΔ enhances autophagy flux in the presence of MAP1S. Lysates were collected from control (MAP1S^+/+^) or MAP1S knockout HeLa cells (MAP1S^−/−^) transiently transfected with HA vector and HA-HBD and HA-HBDΔ in the absence (Ctrl) or presence of BAF. Representative immunoblots (**E**) and quantification of the relative levels of MAP1S in control HeLa cells in the absence of BAF (**F**) or relative levels of LC3-II in the presence of BAF (**G**) are shown. (**H-J**) Overexpression of HBD but not HA-HBDΔ prevents the HDAC4-induced MAP1S destabilization and autophagy flux suppression. Lysates were collected from HeLa cells transiently co-transfected with HDAC4 and HA vector, HA-HBD or HA-HBDΔ in the absence (Ctrl) or presence of BAF. Representative immunoblots (**H**) and quantification of the relative levels of MAP1S in the absence of BAF (**I**) or LC3-II in the presence of BAF (**J**) are shown. (**K-M**) Overexpression of HBD but not HA-HBDΔ reduces levels of mHTT aggregates. N2a cells transiently expressing GFP-HTT74Q were simultaneously transfected with two additional plasmids with one to express control, HBD or HA-HBDΔ, and another to express control or HDAC4, respectively. Equal amounts of cell lysates are subjected to immunoblot. Representative blots of GFP-74Q resolved by stack gel or AGERA (**K**) and their quantification in stack gel (**L**) or AGERA (**M**) are shown. (**N**) A diagram showing the potential mechanism by which HDAC4 regulates MAP1S-mediated autophagy turnover of mHTT aggregates. Under normal condition, isolation membrane-associated acetylated-MAP1S is deacetylated and destabilized by microtubule or aggregate-associated HDAC4. In the presence of excessive HBD, HBD competes with acetylated MAP1S for interaction with HDAC4, which leads to the exposure of mHTT aggregates to be packaged by the MAP1S-associated isolation membrane. The resulted mHTT-containing autophagosomes are connected to microtubules by acetylated MAP1S and fuse with lysosome to become autolysosomes in which mHTT aggregates are degraded by lysosomal enzymes.

To further confirm that the impact of HDAC4 on autophagy and degradation of mHTT aggregates is specifically through its association with MAP1S, we compared the impacts of HBD with HBDΔ on MAP1Smediated autophagy turnover of mHTT aggregates. When either HBD or HBDΔ was overexpressed in HeLa cells, HBD but not HBDΔ reduced the amount of endogenous MAP1S co-precipitated with HDAC4 (Figure 7A,B), suggesting that HBD competed with full length MAP1S for binding with HDAC4. In such a way, overexpression of HBD but not HBDΔ enhanced the stability of endogenous MAP1S (Figure 7C,D) and increased the levels of endogenous MAP1S protein (Figure 7E,F). Consequently, autophagy flux represented by LC3-II levels in the presence of Bafilomycin A1 was enhanced by HBD but not HA-HBDΔ and such enhancive effect was only observed in the presence of endogenous MAP1S (Figure 7E,G). The HDAC4 overexpression-induced deceases in MAP1S levels were prevented when cells expressed HBD but not HAHBDΔ (Figure 7H,I), and HDAC4 overexpression-induced impairment of autophagy flux was reactivated in the presence of HBD but not HA-HBDΔ (Figure 7H,J). Overexpression of HBD but not HA-HBDΔ protected stability of endogenous MAP1S and led to a significant decrease in levels of mHTT aggregates (Figure 7K-M).

Therefore, the HBD interrupts MAP1S-HDAC4 interaction and promotes MAP1S-mediated autophagy turnover of mHTT aggregates (Figure 7N).

## DISCUSSION

HDAC-mediated histone deacetylation is a key epigenetic modification that has attracted enormous attention since histone deacetylases emerged as a druggable class of enzymes [21]. Non-histone protein acetylation has also attracted attention because of the demonstration that histone deacetylase 6 (HDAC6) is involved in microtubule-deacetylation and regulation of autophagy and mitophagy [22–24]. Originally, HDAC4 was characterized as a histone deacetylase regulating transcription factors MEF2 [20] and Runx2 [25]. It was reported that HDAC4 alone does not show deacetylase activity on histone substrate but regulates histone deacetylation through its interaction with HDAC3 [26]. Deletion of HDAC4 in mouse brain was reported to have no effect on histone acetylation profiles and global transcription [27]. It seems that HDAC4 regulates transcription of a specific set of genes by affecting the stability of the related transcriptional factors instead of the general epigenetic modification of genomeassociated histones. Recently, HDAC4 was found to be associated with aggregate-prone mutant Huntington's disease–associated products of the *huntingtin* gene (HTT) that have long polyglutamine stretches [3]. The significance of the HDAC4-huntingtin association has not been assessed at the mechanistic level. Here we present evidence that HDAC4 impairs the degradation of mHTT aggregates, interacts directly with autophagy activator MAP1S, reduces MAP1S stability, consequently suppresses the autophagy flux mediated by MAP1S, and impairs the degradation of mHTT aggregates. Inhibition of HDAC4 resulted in stabilization of MAP1S, activation of MAP1S-mediated autophagy flux and fast degradation of mHTT aggregates. No matter HDAC4 directly or indirectly through HDAC3 exerts its deacetylase activity on MAP1S, this provides a mechanism by which therapeutic reduction of HDAC4-associated activity could reduce accumulation of cytoplasmic mHTT aggregates and ameliorate neurodegeneration [3].

In this study, we implicated and established a mechanism for the role of MAP1S in the development of Huntington's disease through its role in multiple steps of autophagy. Our results show that it is the inhibition of HDAC4 and its associated deacetylase activity that enhances the stability of MAP1S, increases autophagy flux and improves clearance of mHTT aggregates. HDAC4 interacts with mHTT but not wildtype HTT, and is found enriched in mHTT aggregates [3]. Wild-type HTT protein normally is found in association with microtubules [28] and thus may regulate vesicle trafficking in neurons [29]. The homology between regions of HTT and the yeast autophagy regulatory proteins predicted a normal regulatory function in autophagy [30], which has been confirmed [31, 32]. The mHTT disrupted the normal HTT-mediated regulation of autophagosomal dynamics and caused defects in cargo degradation [33]. We reason that soluble mHTT protein may function similar to wild-type HTT in association with motor protein complexes on microtubules. However, mHTT sequesters HDAC4 on microtubules, HDAC4 impairs the stability and microtubule-associated functions of MAP1S through deacetylation and consequently interrupts autophagy flux. This defect leads to accumulation of mHTT and consequent formation of mHTT aggregates. The mHTT aggregates further sequester HDAC4 that further protects the aggregates from degradation by MAP1S-mediated autophagy.

Although there is no major treatment has been developed specifically for Huntington's disease, enhancing clearance of mHTT aggregates has been considered one of the feasible approaches to slow the neurodegeneration in Huntington's disease and has revealed some beneficial effects in animal models [34]. HDAC4 reduction in mouse model by transgenic approach has been shown to delay cytosolic mHTT aggregate formation and improve motor coordination, neurological phenotypes and longevity [3]. This presents a novel strategy to target huntingtin aggregates using small molecules to inhibit HDAC4 activity. Our results provide a detail mechanism for HDAC4 to impact on the degradation of mHTT. Since a series of cellular processes have been reported to be regulated by HDAC4, any small molecule inhibitor of HDAC4 will inhibit the general activity of HDAC4 and is surely expected to cause unnecessary side effect besides the clearance of mHTT aggregates. Here we have identified a HDAC4-binding domain from MAP1S that specifically interrupts the HDAC4-MAP1S interaction and protects MAP1S from being deacetylated by HDAC4-associated deacetylase activity. Thus, further development of short peptides to disrupt the HDAC4-MAP1S interaction will specifically enhance the MAP1S-mediated autophagy clearance of mHTT aggregates.

## METHODS

### Antibodies, siRNAs and other reagents

Antibody against MAP1S (4G1) was a gift from Precision Antibody (AG10006). Antibody against acetylatedtubulin (6–11B-1) was from abcam. Antibodies against HDAC4 (7628), acetylated lysine (9441), Myc (2276) and Huntingtin (2773) were from Cell Signaling Technology. Antibody against HA-tag (MMS-101P) was from Covance. Antibody against acetyl-lysine (05-515) and anti-acetyl lysine agarose conjugate (16–272) were from EMD Millipore. The siRNAs specific to human HDAC4 (sc-35540) and mouse HDAC4 (sc-35541) were from Santa Cruz Biotechnology. Protease inhibitor cocktail and anti-Flag M2-agarose were from Sigma. Other antibodies, siRNAs and reagents were described by Zou et al. [35].

### Plasmids and site-directed mutagenesis

RFP-LC3 was a gift from Dr. Mizushima [36]. HA-MAP1S isoforms (HA-LC, HA-SC, HA-HC and HA-FL) have been described earlier [7]. HA-MAP1S R653-Q855 fragment, representing the HDAC4-binding domain (HBD) of MAP1S was encoded by a PCR-amplified *MAP1S* insert in HA-PCMV plasmid (631604, Clontech Laboratories Inc.). Plasmids encoding GFP–HDAC4, Flag-HDAC4, Flag-HDAC4 H976Y, Flag-HDAC4 H976F, Myc-HDAC4 H803A, or Myc-HDAC4 D840N were previously reported [19, 20, 37]. Plasmid encoding GFP-HTT74Q (#40262) was purchased from Addgene. To delete the HBD fragment from the FL MAP1S to generate the HA-HBDΔ, a pair of primers (5′-CTCGCTGCCCTCTGCGGGGCT-3′ and 5′-ACGGA GAACGTCAGCCGCACC-3′) were phosphorylated with T4 polynucleotide kinase (NEB, M0201) and mixed with HA-FL plasmid template to amplify the HA-HBDΔ by PCR using the KOD hot start DNA polymerase from TOYOBO. The Restriction enzyme *Dpn*I was added to the PCR reaction mixture to digest the template. T4 DNA Ligase (NEB, M0202) was used to ligate the PCR product to become a circular plasmid that was verified by DNA sequence.

### Establishment of stable cell line expressing inducible GFP-HTT72Q

Lenti-X™ Tet-Off^®^ Advanced Inducible Expression System (Clontech, Cat: 632163) was used according to the manufacturer's instruction to generate the inducible GFP-HTT72Q stable cell lines. Briefly, an EGFP-tagged HTT fragment encoded by human *HTT* exon 1 plus 72Q repeat were amplified from the pUAST-Httex1-Q72-eGFP construct as previously described [38] and subcloned into the pLVX-Tight-Puro vector. Lenti-viruses containing Httex1-Q72 plasmid (pLVX-tight-Q72) and the regulator plasmids (pLVX-Tet-OFF advanced) were produced using the Lenti-X HTX packaging system (Clontech) and used to infect HeLa cells as instructed. Puromycin (2 ug/ml) and Neomycin (200 ug/ml) were applied to screen for positive clones, which were being maintained in the “off” state in the presence of 100 ng/ml Dox during the whole selection process to turn off the expression of potentially toxic HTT72Q proteins.

### Establishment of stable MAP1S knockout HeLa cell line by CRISPR/Cas9

Guide RNAs targeting human MAP1S gene were designed using Optimized Crispr Design (http://crispr.mit.edu/). Synthesized DNA oligos were inserted into crispr/cas9 vector pSpCas9(BB)-2APuro (PX459) (Addgene, #48139). HeLa cells were transiently transfected with a pool of five plasmids encoding Cas9 nuclease and guide RNAs targeting for MAP1S or the vector for wild-type control. Cells were selected with 1.5 μg/ml Puromycin starting at 48 hours after transfection. Multiple monoclonal single cell clones were picked and cultured individually in separate wells. Immunoblot analysis of MAP1S levels was used to determine the knockout efficiency. The sequences of DNA oligos for gRNAs are

1. MAP1S g1-F: 5′-CACCGATGGCGGCGGTGGCT GGATC-3′, MAP1S g1-R: 5′-AAACGATCCAGCCA CCGCCGCCATC-3′;
2. MAP1S g2-F: 5′-CACCGTCGTGGTGGGCAGCGA GTTC-3′, MAP1S g2-R: 5′-AAACGAACTCGCTGCC CACCACGAC-3′;
3. MAP1S g3-F: 5′-CACCGGGGCTCCTCACCTACG TCC-3′, MAP1S g3-R: 5′-AAACGGACGTAGGTGA GGAGCCCC-3′,
4. MAP1S g4-F: 5′-CACCGCGGTCTTGGGATGTC GATCC-3′, MAP1S g4-R: 5′-AAACGGATCGACAT CCCAAGACCGC-3′; and
5. MAP1S g5-F: 5′-CACCGTCCACCTCGGTCGAGT GCG-3′, MAP1S g5-R: 5′-AAACCGCACTCGACCG AGGTGGAC-3′.

### Cell transfection and immunoblot analysis

Cell lines used for transfection included HeLa, HEK (human embryonic kidney)-293T, COS7 cells, HeLa cells stably expressing ERFP–LC3 (HeLa-RFP-LC3), N2a, or MEF cells that were established as described [11, 36, 39]. Cell transfection and immunoblot analysis were performed as previously described [35].

### Fluorescent and transmission electron microscopy

HeLa or HeLa-RFP-LC3 cells were fixed and processed for fluorescence microscopy analysis as previously described [35]. For transmission electron microscopy, HeLa cells transfected with Flag-HDAC4 plasmid or HDAC4-specific siRNA were treated with 10 nM BAF for 12 hrs. As previously described [35], cells were fixed and processed for examination with a JEM 1010 transmission electron microscope (JEOL). ImageJ software was used to measure percentages of areas occupied by autophagy vacuoles.

### Analysis of levels of aggregated GFP-HTT-72Q/74Q

As described previously [40], cells were lysed in isolation buffer (50 mM Tris-HCl, pH 8.0, 100 mM NaCl, 5 mM MgCl2, 0.5% NP-40, and protease inhibitors). Insoluble pellets were isolated after 10 min centrifugation at 14000 rpm at 4°C and resuspended in buffer containing 20 mM Tris-HCl, pH 8.0, 15 mM MgCl^2^ and 0.5 mg/ml DNase, and then incubated at 37°C for 1 hr. Insoluble fractions were diluted in loading buffer and boiled for 5 min for immunoblot analysis. Agarose Gel Electrophoresis for Resolving Aggregates (AGERA) was performed following a described protocol [41]. Briefly, 100 μg of total protein was loaded to a 1.5% agarose gels containing 0.1% SDS for AGERA. Gels were run at 100 V and semi-dry transfers were conducted at 200 mA for 1 h. After transfer, PVDF membranes were used for immunoblot analyses.

### Co-immunoprecipitation and GST pull-down assays

Cell lysates were subjected to immunoprecipitation with antibody against MAP1S, HDAC4, or their respective IgG control antibody as described before [35, 42]. For GST pull-down assay, 293T cells overexpressing Flag-HDAC4 or a control were lysed with lysis buffer containing 2 mM Dithiothreitol. Purified GST or GSTtagged proteins (7.5 μg) bound to 25 μl GSH-Sepharose 4B beads [43] were mixed with 200 μg pre-cleaned cell lysates, and each mixture was rotated for 1 hr at 4°C and then washed three times with lysis buffer. The pellets were resuspended in 50 μl of lysis buffer containing loading buffer and boiled for 10 min for immunoblot analyses.

### Assay of HDAC4 deacetylase activity with MAP1S substrates

Lysis buffer for *in vitro* deacetylation assays contained 50 mM HEPES, pH 7.5, 150 mM NaCl, 1 mM EDTA, 2.5 mM EGTA, 0.1% triton X-100 and 10% glycerol. As described previously [44], Flag-HDAC4, Flag-HDAC4 H976Y, Flag-HDAC4 H976F, Myc-HDAC4 H803A, or Myc-HDAC4 D840N was overexpressed in 293T cells and purified with conjugated anti-Flag M2-agarose or anti-Myc antibody and Protein G-agarose beads. HA-MAP1S was over-expressed in 293T cells and purified with anti-MAP1S antibody (4G1). The agarose beads containing immunoprecipitated HDAC4 were mixed with agarose containing HAMAP1S in deacetylation buffer (10 mM Tris-HCl, pH 8.0, 150 mM NaCl and 10% glycerol). Each mixture was incubated at 37°C for an indicated amount of time; each reaction was terminated with loading buffer.

### Detection of MAP1S mRNA levels by quantitative realtime PCR

Total RNA was extracted from HeLa cells transfected with HDAC4-specific siRNA or HDAC4 expression plasmid with Trizol reagent (Invitrogene#15596–026) according to the manufacturer's instructions. The Invitrogene SuperScript III First-Strand System was used for reverse transcription with random primers. Real-time PCRs were performed with SYBR Premix ExTaq (TaKaRa RR820A). Primers for human MAP1S included a forward primer 5′-CGCTGGAAGAACTCCTCATC-3′ and a reverse primer 5′-GAGTGAGCCCAGTGAGAAGG-3′ and those for human β-Actin included a forward primer 5′-ACTCTTCCAGCCTTCCTTCC-3′ and a reverse primer 5′-CAGTGATCTCCTTCTGCATCC-3′. The relative mRNA levels of *MAP1S* were quantified by normalizing the amount of *MAP1S* mRNA to amount of *β-actin* mRNA.

## Abbreviations

AGERA: Agarose Gel Electrophoresis for Resolving Aggregates
BAF: Bafilomycin A1
HBD: HDAC4-binding domain
HBDΔ: MAP1S with HBD deleted
HD: Huntington's disease
HDAC4: histone deacetylase4
K520R: lysine to arginine mutation at amino-acid residue 520
LC3: light chain 3
MAP1S: microtubule-associated protein family 1 small form
mHTT: mutant huntingtin
HTT72Q: Huntingtin gene plus 72 expanded polyglutamine [poly(Q)]
LAMP2: lysosomal-associated membrane protein 2
N2a: Neuroblstoma Neuro-2a
